# Association between hepatitis B vaccine antibody response and CD4 reconstitution after initiation of combination antiretroviral therapy in HIV-infected persons

**DOI:** 10.1186/s12879-015-0937-5

**Published:** 2015-05-01

**Authors:** Kahtonna Allen, Octavio Mesner, Anuradha Ganesan, Thomas A O’Bryan, Robert G Deiss, Brian K Agan, Jason F Okulicz

**Affiliations:** Infectious Disease Clinical Research Program, Uniformed Services University of the Health Sciences, Bethesda, MD USA; Infectious Disease Service, San Antonio Military Medical Center, Fort Sam Houston, TX USA; The Henry M. Jackson Foundation for the Advancement of Military Medicine, Inc, Bethesda, MD USA; Infectious Disease Service, Walter Reed National Military Medical Center, Bethesda, MD USA; Infectious Disease Clinic, Naval Medical Center San Diego, San Diego, CA USA

**Keywords:** HIV, AIDS, Hepatitis B vaccine, Antiretroviral therapy, CD4 cell count

## Abstract

**Background:**

Hepatitis B virus (HBV) vaccine antibody response has been associated with reduced risk of AIDS or death. However, it is unknown whether HBV vaccine responsiveness is associated with improved immune reconstitution during treatment with combination antiretroviral therapy (cART). We evaluated the relationship between HBV vaccine response status and CD4 reconstitution on cART in the U.S Military HIV Natural History Study.

**Methods:**

Participants with viral load <400 copies/mL within 1 year on initial cART and documented HBV vaccination and surface antibody (anti-HBs) prior to cART were included. Participants were characterized as HBV vaccine responders (anti-HBs ≥10 IU/L) or non-responders (<10 IU/L) and further divided into 2 groups based on vaccine administration before or after HIV diagnosis. Linear mixed regression was used to model CD4 reconstitution during the first year of cART.

**Results:**

Of the 307 and 169 participants vaccinated before or after HIV diagnosis, HBV vaccine response occurred in 288 (94%) and 74 (44%), respectively. For those vaccinated before HIV diagnosis, CD4 counts increased by a median 190 [IQR 99–310] cells/mm^3^ for responders and 186 [IQR 116–366] cells/mm^3^ for non-responders during the first year (P = 0.684). Participants vaccinated after HIV diagnosis had median increases of 185 [IQR 76–270] and 143 [IQR 47–238] cells/mm^3^ for responders and non-responders, respectively (P = 0.134). In contrast to those with CD4 > 350 cells/mm^3^ at cART initiation, participants with CD4 < 200 and 200–350 cells/mm^3^ had significantly reduced CD4 gains in both groups by longitudinal mixed models, but there was no difference in CD4 recovery according to HBV vaccine seroresponse.

**Conclusions:**

Although HBV vaccine responsiveness is associated with a reduction in HIV disease progression, HBV vaccine responders do not achieve greater CD4 gains during the first year of cART. Additional clinical markers are needed to predict the magnitude of post-cART immune recovery.

## Background

In the setting of HIV infection, immunization with hepatitis B virus (HBV) vaccine is essential in order to prevent liver-related morbidity and mortality than can occur with HBV co-infection [1,2]. However, HIV-infected patients have diminished vaccine responsiveness compared to HIV-uninfected persons [3-6]. For example, positive seroresponses occur in 20-62% of persons vaccinated after HIV diagnosis compared to approximately 90% in HIV-uninfected individuals. The development of a positive HBV vaccine antibody response involves not only T-cell function but also other functional pathways including B-cell activity and antigen presentation of the peptide-based vaccine [7-9]. Since HBV vaccine seroresponse requires preserved function in a number of immune pathways, the evaluation of vaccine responsiveness in HIV-infected persons may provide useful information about the immune status of the individual beyond measurement of CD4 cell counts.

In a previous study, we reported the risk of developing clinical acquired immune deficiency syndrome (AIDS) or death is 2-fold higher in HBV vaccine non-responders compared to responders after adjusting for HIV disease-related factors such as CD4 count, viral load (VL), and use of combination antiretroviral therapy (cART) [10]. Although HBV vaccine response can predict HIV disease outcomes, it is unknown whether HBV vaccine responders have improved immune recovery during cART. Identifying predictors of CD4 reconstitution during cART is clinically important since the rate of both AIDS and serious non-AIDS events decrease at higher CD4 counts, even among the subgroup of individuals with CD4 counts >500 cells/mm^3^ [11,12]. We retrospectively evaluated the relationship between HBV vaccine response status and post-cART CD4 cell gains in the U.S. Military HIV Natural History Study.

## Methods

The U.S. Military HIV Natural History Study is comprised of over 5700 military beneficiaries with HIV infection as previously described [13]. Participants were ≥18 years of age and provided informed, written consent. Individuals without prior HBV infection who achieved VL suppression, defined as <400 copies/mL within 1 year on their initial cART regimen, and maintained VL suppression for >1 year were included. Participants were divided into 2 groups according to the whether all vaccine doses were received prior to HIV diagnosis or in the interval between HIV diagnosis and cART initiation. Individuals who received HBV vaccine doses both before and after HIV diagnosis were excluded as were individuals vaccinated after cART initiation. Participants were then characterized according to HBV vaccine response, with responders and non-responders defined as having an antibody to HBV surface antigen (anti-HBs) ≥10 or <10 IU/L, respectively ≥30 days after last vaccination. For those vaccinated prior to HIV diagnosis, the first available anti-HBs determination was used to assign participants into responder or non-responder categories. This study was approved by the Uniformed Services University of the Health Sciences Institutional Review Board.

The primary outcome was CD4 cell recovery during the first year of cART in HBV vaccine responders compared to non-responders. Continuous variables were analyzed by *t*-test for normally distributed variables and Wilcoxon for non-normally distributed variables. Normality was assessed using Shapiro-Wilks test. P-values for categorical variables were calculated using chi-squared or Fisher’s exact test when appropriate. To assess and quantify the effect of HBV vaccine response on CD4 reconstitution after cART initiation, all available CD4 counts for participants from cART initiation to one year post-cART were analyzed. To quantify the adjusted effect of vaccine response on the rate of CD4 increase after cART, longitudinal mixed models were performed which allowed use of all CD4 values in the window of interest while accounting for within participant CD4 association. The model adjusts for race, gender, age at cART initiation, AIDS before cART, and HBV vaccine response status at baseline as well as CD4 at cART and vaccine response status on CD4 gains after cART. Power to detect differences among variables was enhanced by multiple CD4 count measurements contributed per participant. Lastly, sensitivity analyses were performed for several variables, including year of cART initiation, and time from HIV diagnosis to cART initiation. Additional variables were used to assess effect modification post hoc, however none of these variables were significant modifiers for the outcome of interest.

## Results

A total of 307 participants met criteria for HBV vaccination prior to HIV diagnosis, including 288 (94%) responders and 19 (6%) non-responders (Table 1). Demographic characteristics were similar between groups except the responder group had a higher proportion of African Americans. The median CD4 count at cART initiation was 360 [IQR 270–448] cells/mm^3^ compared to 298 [IQR 234–394] cells/mm^3^ for responders versus non-responders (P = 0.398), respectively and VL was similar between groups. Participants vaccinated prior to HIV diagnosis had a median of 4 (IQR 2–5) CD4 count determinations during the first year on cART and a median of 3 (IQR 2–5) values were available for those vaccinated between HIV diagnosis and cART initiation. Regimens commonly included non-nucleoside reverse transcriptase inhibitors (NNRTIs) in both groups, however the median year of cART initiation was later for responders compared to non-responders (2008 vs 2003; P = 0.051).

**Table 1:** **Baseline characteristics for participants vaccinated before or after HIV diagnosis**

	**Vaccinated Before HIV Diagnosis**				**Vaccinated After HIV Diagnosis**			
**Characteristic**	**Total**	**Vaccine responder**	**Vaccine non-responder**	**P value**	**Total**	**Vaccine Responder**	**Vaccine non-responder**	**P value**
Number, n	307	288 (94)	19 (6)	--	169	74 (44)	95 (56)	--
Age at HIV diagnosis (years)	29 (25-36)	29 (25-36)	34 (26-41)	0.170	28 (24-34)	27 (24-32)	29 (25-34)	0.349
Gender, Male	291 (95)	274 (95)	17 (89)	0.260	148 (94)	63 (85)	85 (89)	0.540
Ethnicity				0.085				0.142
Caucasian	116 (38)	104 (36)	12 (63)		70 (41)	28 (38)	42 (44)	
African American	134 (44)	129 (45)	5 (26)		69 (41)	28 (38)	41 (43)	
Hispanic/Other	57 (18)	55 (19)	2 (11)		30 (18)	18 (24)	12 (13)	
Median CD4 Count (cells/mm^3^) at HIV diagnosis	436 (307-570)	438 (312-570)	372 (282-561)	0.335	526 (405-711)	543 (422-724)	517 (382-676)	0.129
Median VL at HIV diagnosis (log_10_ copies/mL)	4.5 (3.9-5.0)	4.5 (3.9-5.0)	4.7 (4.2-5.1)	0.853	4.2 (3.5-4.4)	4.2 (3.4-4.6)	4.2 (3.5-4.4)	0.888
Number of HBV vaccine doses	3 (2-3)	3 (1-3)	3 (3-3)	0.101	3 (1-3)	2 (1-3)	3 (2-3)	0.585
Median time from last vaccination to anti-HBs determination (months)	18.1 (6.1-57)	26 (7.4-67)	6.8 (4.3-15)	0.222	5.2 (2.8-6.8)	5.5 (2.8-7.1)	4.9 (2.7-6.4)	0.340
Median CD4 Count at last HBV vaccination (cells/mm^3^)	--	--	--	--	489 (353-628)	481 (331-621)	510 (394-629)	0.669
Median VL at last HBV vaccination (log_10_ copies/mL)	--	--	--	--	4.1 (3.6-4.6)	4.1 (3.7-4.6)	4.1 (3.3-4.4)	0.274
Year of cART initiation	2008 (2003-2010)	2008 (2003-2010)	2003 (2000-2008)	0.051	1998 (1997-2006)	2004 (1997-2009)	1998 (1997- 2004)	0.001
Median time from HIV diagnosis to cART initiation (months)	7.8 (2.0-27.0)	8 (2.1-29.0)	2.1 (1.3-11.0)	0.273	57 (29-91)	51 (29-93)	59 (29-90)	0.603
Median CD4 Count (cells/mm^3^) at cART initiation	356 (270-445)	360 (270-448)	298 (234-394)	0.398	379 (249-473)	390 (277-492)	377 (215-460)	0.137
Median VL at cART initiation (log_10_ copies/mL)	4.5 (4.0-5.0)	4.5 (3.9-5.0)	4.4 (4.3-5.0)	0.619	4.4 (3.6-4.8)	4.4 (3.9-4.7)	4.4 (3.5-4.8)	0.864
Initial cART regimen				0.669				0.612
NNRTI	193 (63)	180 (62)	13 (68)		67 (40)	31 (42)	36 (38)	
Boosted PI	34 (11)	31 (11)	3 (16)		18 (11)	5 (7)	13 (14)	
Unboosted PI	52 (17)	49 (17)	3 (16)		67 (40)	29 (39)	38 (40)	
Triple NRTI	26 (8)	26 (9)	0 (0)		7 (4)	4 (5)	5 (5)	
Other	2 (1)	2 (1)	0 (0)		10 (6)	5 (7)	0 (0)	
Hepatitis C diagnosis before cART initiation	9 (3)	9 (3)	0 (0)	1.000	8 (5)	3 (4)	5 (5)	1.000

HBV, hepatitis B virus; responder, antibody to HBV surface antigen (anti-HBs) ≥10 IU/L; non-responder, anti-HBs <10 IU/L; VL, viral load; cART, combination antiretroviral therapy; NNRTI, non-nucleoside reverse transcriptase inhibitor; PI, protease inhibitor; NRTI, nucleoside reverse transcriptase inhibitor.

Data are number (%) or median (interquartile range) unless otherwise specified.

For participants vaccinated after HIV diagnosis but prior to cART initiation (n = 169), 74 (44%) were responders and 95 (56%) were non-responders. Demographic and vaccine-related characteristics were similar between groups, including the number of vaccine doses, and CD4 and VL at the time of last vaccination. At cART initiation, the median CD4 count (390 [IQR 277–492] vs 377 [IQR 215–460] cells/mm^3^; P = 0.137) and median VL (4.4 [IQR 3.9-4.7] vs 4.4 [IQR 3.5-4.8] log_10_ copies/mL; P = 0.864) were not different for responders compared to non-responders, respectively. Responders initiated cART at a later median calendar year compared to non-responders (2004 vs. 1998; P = 0.001) and the majority of participants in both groups received protease inhibitor (PI)-based regimens.

Among those vaccinated before HIV diagnosis, the median CD4 cell gains 1 year post-cART were similar for HBV vaccine responders compared to non-responders (190 [IQR 99–310] cells/mm^3^ vs. 186 [IQR 116–366] cells/mm^3^; P = 0.684, respectively). For participants vaccinated after HIV diagnosis, vaccine responders had greater CD4 gains (185 [IQR 76–270] cells/mm^3^) compared to non-responders (143 [IQR 47–238] cells/mm^3^), however the difference was not statistically significant (P = 0.134). Longitudinal mixed models for CD4 gains during the first year of cART (Table 2) showed that CD4 count <200 cells/mm^3^ at cART initiation was associated with reduced CD4 gains for those vaccinated before (−208 cells/mm^3^, 95% CI −283, −134; P < 0.001) and after HIV diagnosis (−188 cells/mm^3^, 95% CI −295, −81; P < 0.001). A similar effect was also observed for participants with pre-cART CD4 counts between 200–350 cells/mm^3^ for those vaccinated before or after HIV diagnosis (−141 cells/mm^3^, 95% CI −189, −94; P < 0.001 and −81 cells/mm^3^, −167, 5; P = 0.067, respectively). HBV vaccine responder status was not associated with greater CD4 recovery in those vaccinated either before (−18 cells/mm^3^, 95% CI −79, 42; P = 0.551) or after HIV diagnosis (26 cells/mm^3^, 95% CI −55, 108; P = 0.528).

**Table 2:** **Modeled CD4 gains after cART initiation for participants vaccinated before or after HIV diagnosis**

	**Median [IQR] Change in CD4 Cell Count (cells/mm** ^**3**^ ) 1 Year After cART Initiation*			
**Characteristic**	**Vaccinated before HIV diagnosis**	**P value**	**Vaccinated between HIV diagnosis and cART Initiation**	**P value**
CD4 <200 cells/mm^3^at cART initiation	−208 [−283, −134]	<0.001	−188 [−295, −81]	<0.001
CD4 200–350 cells/mm^3^ at cART initiation	−141 [−189, −94]	<0.001	−81 [−167, 5]	0.067
HBV vaccine responder at cART initiation	−18 [−79, 42]	0.551	26 [−55, 108]	0.528

cART, combination antiretroviral therapy; IQR, interquartile range; HBV, hepatitis B virus; responder, antibody to HBV surface antigen ≥10 IU/L.

*Model adjusted for HBV vaccine response status, race, gender, age at cART initiation, and AIDS before cART at baseline.

## Discussion

In addition to the importance of HBV vaccine in the prevention of HBV infection, positive vaccine response has been associated with improved HIV prognosis. In a previous study, we found that 22% of HBV vaccine non-responders developed AIDS or death compared to 5% of responders (P < 0.001) [10]. Based on these findings, we studied whether the beneficial functional immune aspects inherent in vaccine responders translated to more robust immune responses after initiation of VL-suppressive cART. Characteristics associated with differential CD4 gains on cART in previous studies were no different among our participant groups, including age, regimen type, duration of HIV infection, and pre-cART CD4 counts and VL [14-16]. In accordance with prior studies in our cohort [13,17], we observed that CD4 gains were blunted in those with lower CD4 cell counts at cART initiation, particularly in those with CD4 counts <200 cells/mm^3^. However, the magnitude of CD4 gains was comparable for HBV vaccine responders compared to non-responders regardless of whether participants were vaccinated before or after HIV diagnosis. Although HBV vaccine seroresponse did not predict CD4 reconstitution after cART initiation, response status remains an important tool as a reduction in AIDS or death events was observed in vaccine responders independent of CD4 cell count in our prior study, including those with CD4 counts >500 cells/mm^3^.

The immunity represented by CD4 count may not be fully indicative of immune functions that lead to the production of anti-HBs antibodies at an effective titer. In addition to our current study examining CD4 outcomes by HBV vaccine seroresponse, studies of other HIV prognostic markers demonstrated similar findings. For example, delayed-type hypersensitivity (DTH) response to recall antigens, an assessment of cell-mediated immunity, has been associated with HIV disease progression [18,19]. However, pre-cART DTH responsiveness did not predict CD4 gains during VL-suppressive cART [20]. Discordance between CD4 count and HIV clinical outcomes was observed in the SILCAAT and ESPIRIT studies [21]. In these clinical trials, infusion of interleukin-2 (IL-2) in addition to cART resulted in larger, sustained CD4 gains compared to cART alone. However, the increase in CD4 count did not translate to improved clinical outcomes as measured by development of opportunistic infections or death, as there was no difference in these outcomes in those treated with cART plus IL-2 compared to cART alone. The observation of nearly identical CD4 gains for HBV vaccine responders and non-responders during the first year of cART in our current study may be explained by differing pathways involved in CD4 reconstitution during cART compared to those responsible for development of HBV vaccine seroresponse.

The strengths of this study include a population with early enrolment after HIV infection and free access to care, as these can be confounders in other cohorts. Based on the composition of our military cohort, our findings may not be applicable to women or those with a history of intravenous drug use or HCV co-infection. The magnitude of anti-HBs titers were not examined as quantitative titers were not routinely performed in clinical practice. Other limitations include the retrospective study design and non-randomized administration of HBV vaccine either before or after HIV diagnosis. The vaccine manufacturer and dose administered were not recorded, however there are conflicting data regarding the efficacy of high-dose HBV in HIV-infected persons [22,23]. The decision to initiate cART was non-randomized and at the discretion of individual providers and patients. Although cART regimens were compared by drug class, specific regimens could not be directly compared due to sample size constraints.

## Conclusions

We observed no difference in CD4 gains for HBV vaccine responders compared to non-responders by the examination of CD4 gains at 1 year post-cART and by longitudinal mixed modeling of all CD4 values during the first year of therapy. This lack of effect was observed in those vaccinated before or after HIV diagnosis. Since HBV vaccine responsiveness has been associated with reduced risk of AIDS or death, it is possible that vaccine responders may have improved immune recovery during cART that is not measured by CD4 counts. For example, T-cell activation and exhaustion, which have both been associated with HIV disease progression and death, may continue to have detrimental effects during VL-suppressive cART [24,25]. The development and validation of other clinically relevant immune correlates are necessary to better predict both HIV progression and immune recovery during cART.

## Abbreviations

HBV: Hepatitis B virus
AIDS: Acquired immune deficiency syndrome
VL: Viral load
cART: Combination antiretroviral therapy
Anti-HBs: Antibody to HBV surface antigen
NNRTI: Non-nucleoside reverse transcriptase inhibitor
PI: Protease inhibitor
DTH: Delayed-type hypersensitivity
IL-2: Interleukin 2
